# Blood biomarkers as surrogate endpoints of treatment responses to aerobic exercise and cognitive training (ACT) in amnestic mild cognitive impairment: the blood biomarkers study protocol of a randomized controlled trial (the ACT Trial)

**DOI:** 10.1186/s13063-019-3798-1

**Published:** 2020-01-06

**Authors:** Danni Li, Michelle M. Mielke, W. Robert Bell, Cavan Reilly, Lin Zhang, Feng Vankee Lin, Fang Yu

**Affiliations:** 1 Department of Lab Medicine and Pathology, University of Minnesota, 420 Delaware Street SE, MMC 609, Minneapolis, MN 55455 USA; 2 Department of Neurology and Health Sciences Research, Mayo Clinic College of Medicine, Rochester, MN 55902 USA; 3 Division of Biostatistics, School of Public Health, University of Minnesota, Minneapolis, MN 55455 USA; 4 University of Rochester Medical Center, 601 Elmwood Ave, Rochester, NY 14642 USA; 5 School of Nursing, University of Minnesota, 5-140 WDH, 308 Harvard St SE, Minneapolis, MN 55455 USA

**Keywords:** Aerobic exercise, Alzheimer’s disease, Cognitive training, Blood biomarkers, Neuropathological biomarkers, Neurodegenerative biomarkers, Neurotrophic biomarkers, Surrogate endpoints, Treatment responses and amnestic mild cognitive impairment

## Abstract

**Background:**

Alzheimer’s disease (AD) is an epidemic with tremendous public health impacts because there are currently no disease-modifying therapeutics. Randomized controlled trials (RCTs) for prevention of AD dementia often use clinical endpoints that take years to manifest (e.g., cognition) or surrogate endpoints that are costly or invasive (e.g., magnetic resonance imaging [MRI]). Blood biomarkers represent a clinically applicable alternative surrogate endpoint for RCTs that would be both cost-effective and minimally invasive, but little is known about their value as surrogate endpoints for treatment responses in the prevention of AD dementia.

**Methods:**

The objective of this study is to investigate blood neuropathological, neurodegenerative, and neurotrophic biomarkers as surrogate endpoints for treatment responses to three interventions in older adults with amnestic mild cognitive impairment (aMCI, a prodromal stage of AD): aerobic exercise, cognitive training, and combined aerobic exercise and cognitive training (ACT). We chose these three sets of biomarkers for their unique mechanistic associations with AD pathology, neurodegeneration and neurogenesis. This study is built on the ACT Trial (1R01AG055469), a single-blinded, multi-site, 2 × 2 factorial phase II RCT that examines the synergistic effects of a 6-month ACT intervention on cognition and MRI biomarkers (AD-signature cortical thickness and hippocampal volume) (*n* = 128). In this ACT Trial blood biomarkers study, we will enroll 120 ACT Trial participants with aMCI and measure blood biomarkers at baseline and at 3, 6, 12, and 18 months. The goals are to (1) determine the effect of interventions on blood biomarkers over 6 months, (2) evaluate blood biomarkers as surrogate endpoints for predicting cognitive responses to interventions over 18 months, and (3, exploratory) examine blood biomarkers as surrogate endpoints for predicting brain MRI biomarker responses to interventions over 18 months.

**Discussion:**

This study aims to identify new blood biomarkers that can track cognitive decline or AD-related brain atrophy among patients with aMCI subjected to a regimen of aerobic exercise and cognitive training. Findings from this study will drive the further use of blood biomarkers in developing effective prevention and treatment strategies for AD dementia.

**Trial registration:**

ClinicalTrials.gov, NCT03313895. Registered on 18 October 2017.

## Background

In 2018, 5.7 million living Americans were known to have a diagnosis of Alzheimer’s disease (AD) dementia, and 13.8 million Americans will be affected by 2050 [1]. The economic toll of AD is high, estimated at $277 billion in 2018 and is projected to increase to $1.1 trillion by 2050 (in 2018 dollars) [1]. Up to the present time, no viable therapies exist to prevent or cure AD [2]. A successful intervention that delays AD onset by 5 years could save $89 billion a year in the USA alone [1]. However, there are issues in selecting the appropriate study endpoints for evaluating efficacy of AD prevention trials because cognitive decline in amnestic mild cognitive impairment (aMCI, the prodromal stage of AD) or pre-clinical AD takes years or even decades to manifest. Biomarkers could address this problem and are widely used as surrogate endpoints in other conditions (e.g., blood cholesterol for monitoring lipid-lowering therapy in the prevention of cardiovascular diseases) [3].

In AD, only neuroimaging and cerebrospinal fluid (CSF) biomarkers have been proposed as surrogate endpoints, but they are both costly and/or invasive. Blood biomarkers predictive of treatment responses would be more advantageous than neuroimaging and CSF biomarkers due to their broad clinical applications. Three sets of blood biomarkers have been well studied in AD. The first set includes blood neuropathological biomarkers (e.g., decreased amyloid beta 42 [Aβ_42_] or ratio of Aβ_42_/Aβ_40_ and increased phosphorylated tau [p-tau]) that differentially reflect AD pathology [4, 5] and associated neurodegeneration [6], which have also been demonstrated to be associated with cognitive impairment or dementia [6, 7]. The second set includes blood neurodegenerative biomarkers total tau (t-tau) and neurofilament light (NfL). Both have shown to be promising as a biomarker of neuronal injury [8]. Increased baseline plasma t-tau levels have correlated with greater cognitive decline in older adults with MCI over time [9]. NfL has been tested as a surrogate endpoint for treatment responses in a neurological condition (multiple sclerosis) [10]. The third set includes blood neurotrophic biomarkers (e.g., brain-derived neurotrophic factor [BDNF], insulin-like growth factor 1 [IGF-1], and short-chain acylcarnitines [SCACs]) that are found to promote neurogenesis [11, 12] and protect against cognitive decline [13, 14] and incident AD [15, 16]. Higher plasma BDNF levels were linked to better memory, larger hippocampal volume, and reduced risk for AD [11, 15, 17]. A recent study showed that higher plasma levels of IGF-binding protein 3 (IGFBP-3), a protein essential for IGF-1 physiological action [18], were associated with decreased risk for dementia [16]. Our own study further linked higher levels of plasma SCACs (dl-carnitine [C0] and valeryl-l-carnitine [C5]) to less cognitive decline over 7 years in older adults with normal cognition [19]. Studies have examined whether changes in these biomarkers are associated with changes in cognition or in brain magnetic resonance imaging (MRI) biomarkers among people with aMCI [9]. Hence, these blood biomarkers have considerable potential as surrogate endpoints for treatment response in aMCI.

Aerobic exercise and cognitive training are promising lifestyle interventions in the context of AD. However, the mechanisms of action between these interventions differ. Aerobic exercise is hypothesized to induce widespread and permanent molecular and cellular changes that underlie both neurodegeneration and neurogenesis [20–22]. In contrast, cognitive training is hypothesized to induce minor brain structural changes but contribute relatively strong functional changes in trained cognitive domains [23–25]. Furthermore, combined aerobic exercise and cognitive training (ACT) may have a synergistic effect on cognition [26]. However, little is known as to if and how the respective or combined effects of ACT will impact blood neuropathological, neurodegenerative, and neurotrophic biomarkers.

### Study aims

The objective of this study is to investigate blood neuropathological, neurodegenerative, and neurotrophic biomarkers as surrogate endpoints for treatment response to three interventions in older adults with aMCI: aerobic exercise, cognitive training, and ACT. The specific aims and hypotheses of the study are described as follows:

- Aim 1: Determine the effect of interventions on blood biomarkers over 6 months in aMCI.
  *-- Hypothesis 1a:* ACT decreases t-tau, p-tau, and NfL and increases Aβ_42_ (or the ratioAβ_42_/Aβ_40_), BDNF, IGF-1, and SCACs to a greater extent than aerobic exercise or cognitive training alone.
  *-- Hypothesis 1b (primary):* Aerobic exercise induces favorable changes in three sets of biomarkers, while cognitive training only causes favorable changes in neurotrophic biomarkers.
- Aim 2: Evaluate blood biomarkers as surrogate endpoints for predicting cognitive response to interventions over 18 months in aMCI.
  *-- Hypothesis 2*: Greater favorable changes in blood biomarkers at 3 and 6 months are associated with more favorable cognitive changes at 6 months and at 12 and 18 months, respectively.
- Aim 3 (exploratory): Examine the correspondence between changes in blood and MRI biomarkers in response to interventions over 18 months in aMCI. If intervention effects on blood and MRI biomarkers can be correlated, it will provide further evidence that blood biomarkers are valuable surrogate endpoints in aMCI.

## Methods

### Design

**Fig. 1:**
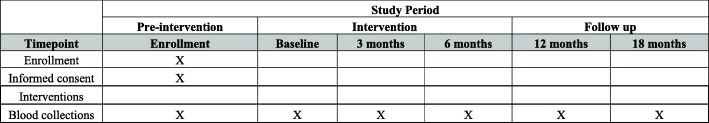
Schedule of enrollment, interventions, and blood collections

This blood biomarker study is built on the design of the ACT Trial, which is a single-blinded (participants are blinded to their treatment groups), two-site (University of Minnesota [UMN] and the University of Rochester [UR]), 2 × 2 factorial randomized controlled trial (RCT) to test the efficacy and synergistic effects of a 6-month ACT regimen on cognition and relevant mechanisms (aerobic fitness, cortical thickness, and functional connectivity in the default mode network) in older adults (age 65 years and older) with aMCI [26]. Details of the ACT Trial have been published [26]. The ACT Trial will randomize 128 participants equally to 4 arms (*n* = 32 per arm): aerobic exercise (cycling) only; speed of processing (SOP) cognitive training only; ACT; or attention control for 6 months. All participants will then be followed for another 12 months. Cognition, including executive function and episodic memory, will be assessed at baseline and at 3, 6, 12, and 18 months. MRI biomarkers (AD-signature cortical thickness and hippocampal volume) will be assessed at baseline and at 6, 12, and 18 months. Because the ACT Trial does not collect blood samples, our ancillary study will enroll ACT Trial participants, perform blood collections, and measure blood biomarkers at baseline and at 3, 6, 12, and 18 months (Fig. 1). The ACT Trial is registered with ClinicalTrials.gov (NCT03313895). All items from the World Health Organization Trial Registration Data Set can be found in the ACT Trial’s registration.

Prior to any recruitment, the ACT Trial statistician created a randomization schedule in Research Electronic Data Capture (REDCap), using a random number generator. Randomization was stratified by age (65–74, > 75) and site (UMN, UR) in random blocks of 4 or 8. After completing baseline data collection, participants were randomized. The ACT Trial project manager then logged into the REDCap randomization module and revealed the participant’s group allocation. The project manager is the only person who can access the randomization module. The ACT Trial investigators and data collectors are blinded to group allocation, and participants are reminded to not discuss their assigned activity with data collectors, who also re-emphasize with the participants the importance of not discussing their activities during assessments. Under no circumstances will the ACT Trial reveal allocation, and unblinding is not permissible [26]. Also, this ancillary study will not have access to the intervention assignments and will not reveal allocation or unblinding.

### Interventions

Participants started their assigned activities (cycling only, SOP training only, ACT, or attention control) within 2 weeks of completing baseline data collection. Each activity includes three weekly, supervised sessions for 6 months or 72 total sessions delivered over 28 weeks, which accounts for missing sessions (e.g., due to illness and vacation). One interventionist supervises two to three participants per session. Cycling only includes moderate-vigorous intensity as 50 to 75% of heart rate (HR) reserve and/or 11–15 on the Borg Rating of Perceived Exertion (RPE) scale for 30–50 min per session. Cycling is set at 50–60% of HR reserve or RPE 11–12 for 30 min in session 1, and is increased by 5% of HR reserve (or 1 point on Borg) or 5-min increments as tolerated up to 65–75% of HR reserve (or RPE 13–15) for 50 min per session over time. For the SOP training, the ACT Trial uses the InSight online program (Posit Science) consisting of five games (Eye for Detail, Hawk Eye, Visual Sweeps, Double Decision, and Target Tracker). These SOP training games target multiple cognitive processes, primarily in attention and processing speed, and become increasingly difficult and require faster reaction times. In the ACT group, the participants cycle first and play SOP games second without a time lapse. In the attention control group, participants stretch (i.e., seated movements and static stretches) [27] and engage in mental leisure activities (MLAs) (i.e., online word search, Sudoku, and solitaire games) [23, 27] that are matched to session durations for cycling and SOP training groups, respectively. These activities serve two purposes: (1) they are controls of the social interaction, computer, and online experience effects of the ACT; and (2) they increase participant retention.

Data entry research assistants, statisticians, the Principal Investigators (PIs), the study coordinators, and the ACT Trial Data Safety and Monitoring Board (DSMB) will monitor integrity and validity of the collected data and ensure participants’ safety. The composition and reporting structure of the ACT Trial’s DSMB has been published previously [26]. All study-related unanticipated adverse events (AEs) and any serious AEs are reported to the Institutional Review Board (IRB), DSMB, and National Institute on Aging (NIA), the ACT Trial study sponsor, within 5 business days of the event. Expected AEs are outlined in the consent forms and are not reported to the IRB unless they persist. The DSMB in conjunction with the NIH office would make the final decision to terminate the trial.

The ACT Trial may terminate study participation temporarily or permanently based on medical circumstances as well as psychological and/or behavioral symptoms. If a participant requests to withdraw, the ACT Trial would terminate all study-related activities immediately. During the course of the trial, the ACT Trial does not limit what the study participants can or cannot do, except for enrolling in another clinical trial which may affect cognition. There are no provisions (e.g., ancillary and post-trial care) or compensation to participants who suffer harm from trial participation.

### Settings

Blood sample collections and processing will be performed using the same standardized protocols at both study sites. All samples will be stored in the − 80 °C freezer in PI Li’s lab. We will perform biochemical analyses of blood biomarkers at the UMN under PI Li’s supervision. All personnel involved in blood sample collection, processing, storage, and biomarker biochemical assessment will be blinded to participants’ ACT Trial treatment arm assignment.

### Study population

#### Recruitment and consent

**Fig. 2:**
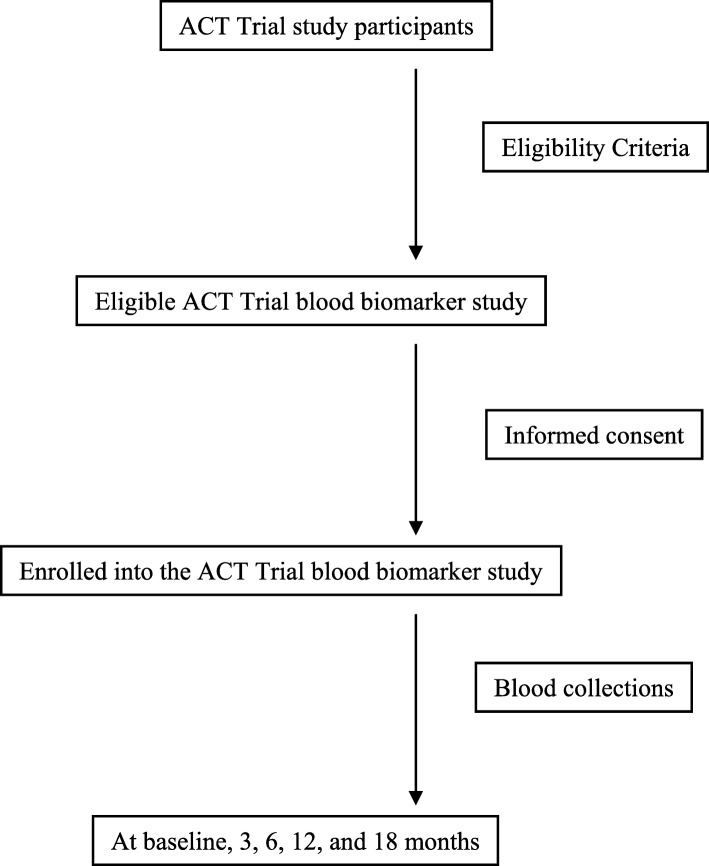
Schedule of enrollment, interventions, and blood collections

Recruitment for this ancillary study will begin as soon as the ACT Trial initiates enrollment of its participants. Participants meeting the ACT Trial eligibility criteria [26] will need to meet two additional requirements to be eligible for the ancillary study: (1) they agree to donate 20 mL at each blood collection (100 mL total), and (2) they agree to fast for at least 8 h (no food or drink other than water and prescribed medicines) before blood collection. The ACT Trial project managers will recruit and screen participants for eligibility and consent to the ancillary study. We will only enroll participants who have consented to be in this study. We will be blinded to participants’ treatment arm in the ACT Trial, and we will not seek to balance the participants in the blood biomarker study in terms of ACT Trial treatment arms. Figure 2 illustrates the flow chart of the study process.

#### Sample size and power

All 128 ACT Trial participants are expected to meet the preceding two eligibility criteria. We expect to enroll 94% of ACT Trial participants in this ancillary study and have the same attrition rates as the ACT Trial: 25% at 6 months, 30% at 12 months, and 35% at 18 months. As a result, our sample sizes will be 120 at baseline, 90 at 6 months, 84 at 12 months, and 78 at 18 months. Our study is a pilot study and will provide preliminary results to estimate effect sizes for power calculations in a future large-scale study. We evaluated power for all three aims using a total sample size of 90 at 6 months and uneven distribution of sample sizes across 4 arms (with the difference in sample size between any 2 arms no greater than 3). For Aim 1, we conducted power calculations based on 5000 Monte Carlo simulations for the primary hypothesis (*Hypothesis 1b*), which is to test the main effects of ACT on blood biomarker levels at 6 months. With a sample size of 90, Aim 1 has 86% power to detect a moderate main intervention effect (Cohen’s *d* of 0.9). Mean plasma NfL levels were decreased by a Cohen’s *d* of 0.9 in response to drug therapy in a neurological condition (multiple sclerosis) [10]. Therefore, a Cohen’s *d* of 0.9 is realistic and achievable for neurological conditions such as MCI and AD. For Aim 2, we want to test the hypothesis of associations between blood biomarker changes and cognitive changes over time using linear regression models, which have the cognitive changes as the outcomes, and the blood biomarker changes (in log scale) as well as interventions, age, sex, and a binary indicator for the presence of the *APOE* ε4 allele as the predictors. We calculated powers using the R package ‘powerMediation’. Aim 2 has more than 80% power to detect a weak correlation (*r* = 0.3) at a significance level of 0.05. Exploratory Aim 3 will have the same power as for Aim 2.

#### Retention

The ACT Trial will use 16 retention and adherence strategies: 10 at the program level to improve safety, enjoyment, comfort, and convenience, and 6 at the staff level to enhance communication, rapport, sympathy, and encouragement [26]. We further contribute to recruitment, enrollment, retention, and adherence in both studies by employing five additional strategies to address the procedural concerns: training skilled phlebotomists for blood collection to ensure safety and comfort, conducting phone calls after blood collection to address concerns and ensure future participation, utilizing flexible scheduling of blood collection, providing transportation to blood collection, and compensating participants for blood collection.

### Assessment of blood biomarkers

#### Blood sample collection and processing

We will collect blood samples after but within 2 weeks as the corresponding cognitive assessments at baseline and at 3, 6, 12, and 18 months of the ACT Trial. Importantly, we will implement the following rules in blood collection in order to reduce pre-analytical variations that could affect biomarker levels: (1) collect blood samples after at least 8 h of fasting (only water and medications are allowed), at the same time in the morning (between 8:30 am and 10 am) [28], and after the participant has been sitting for at least 10 min; (2) obtain information on medications, infection, vascular disease conditions, and diets [7]; and (3) collect blood at least 24 h after the last intervention session [29, 30] at 3 and 6 months, in order to mitigate any effects on biomarkers (e.g., IGF-1 and BDNF) from the last bout of intervention.

The ACT Trial project managers at the UMN and UR will schedule the blood collections. Before collection, the ACT Trial project managers and staff will complete a blood-draw assessment designed to record information (e.g., infection, vascular disease conditions, and diets) that may affect blood biomarkers. The day before the blood collection, the project managers remind participants by phone of their appointments and the fasting requirement. On the day of blood collection, trained phlebotomists will collect blood samples following a venous-blood collection protocol. If a participant has forgotten to fast, the phlebotomist will notify the project managers, who will reschedule the blood draw. The phlebotomist will collect a total of 20 mL of blood, half into a 10-mL plasma ethylenediamine tetraacetic acid (EDTA)-treated tube and the other half into a 10-mL serum tube. At the UMN, blood collections will be performed by trained phlebotomists at the study participants’ homes. Upon collection, blood specimens will be stored immediately on wet ice and transported to PI Li’s lab at the UMN for processing. A lab technician will process and aliquot these specimens according to an established protocol. Briefly, the plasma and serum tubes will be gently mixed and centrifuged at 4 °C using a temperature-controlled centrifuge with a swing-out rotor at 1439 *g* for 15 min. The tubes will be removed from the centrifuge immediately after completion. From the plasma tube, up to eight plasma aliquots of 500 μL each will be made; from the serum tube, up to six serum aliquots of 500 μL each will be made; packed cells from each plasma tube will be transferred into a 2-mL aliquot. The aliquoted samples (i.e., plasma, serum, and packed cells separated from the plasma) will be stored in an − 80 °C freezer in PI Li’s lab. At the UR, blood specimens will be performed and processed by staff using the same blood collection and processing protocols and stored temporarily in an − 80 °C freezer locally before being shipped to UMN to PI Li’s lab for long-term storage at UMN.

#### Biochemical analyses

We will perform biochemical analyses of the following biomarkers: (1) plasma neuropathological and neurodegenerative biomarkers Aβ_42_, Aβ_40_, t-tau, p-tau, and NfL; (2) serum neurotrophic biomarkers BDNF and IGF-1 (IGFBP-3 is included as part of the IGF-1 evaluation) [16]; (3) plasma neurotrophic biomarker SCACs; (4) *APOE* genotypes, at the end of the study to minimize the overall variance in biochemical analyses. The biochemical methods are described as follows:

- *Plasma Aβ*_*42*_*, Aβ*_*40*_*, t-tau, p-tau, and NfL*. Single-molecule enzyme-linked immunosorbent assay (SiMoA) is an ultra-sensitive method coupled with the HD-1 analyzer to measure blood protein biomarkers [31] with high precision [7] and elimination of the matrix interferences reported with traditional enzyme-linked immunosorbent assays (ELISAs) for measurement of Aβ_42_ [32, 33]. SiMoAs have been recently used in epidemiological studies to measure blood neuropathological biomarkers [7, 10, 34–36]. We have used a SiMoA to measure plasma t-tau [9]. In this study, we will use commercially available SiMoAs to measure Aβ_42_, Aβ_40_, t-tau, p-tau, and NfL in plasma samples.
- *Serum BDNF, IGF-1, and IGFBP-3*. In this study, we will use commercially available ELISAs (R&D Systems, Minneapolis, MN, USA) to measure BDNF, IGF-1, and IGFBP-3 in the serum samples [37–39].
- *Plasma SCACs*. We have used the Biocrates p180 kits to measure plasma metabolites [19, 40]. In this study, we will use the p180 kits to measure two SCACs, C0 and C5, in the plasma samples.
- *APOE genotypes*. We will extract DNA from packed cells stored at − 80 °C using Puregene® reagents (Qiagen, Germantown, MD, USA) and determine *APOE* genotypes (ε2/ε2, ε2/ε3, ε3/ε3, ε3/ε4, ε2/ε4, or ε4/ε4) using TaqMan® SNP Genotyping Assays for rs429358 and rs7412 (Life Technologies, Carlsbad, CA, USA).

### Data management

This ancillary study does not need a data monitoring committee, as the venous-blood collection presents minimal risk to participants’ safety, and the integrity and validity of the biochemical data are ensured by a Data Quality Monitoring Plan, for which the PI of this ancillary study is responsible. In this plan, the PI, the ACT Trial managers, and the lab study coordinator and technicians monitor and oversee all the study records, adherence to the protocols, number of blood collections performed, and number of samples received, aliquoted, and stored. All paper and electronic forms are identified only with a unique ID number to protect the privacy and safety of the subjects. Paper copies of data and records at the UMN and UR are stored in a dedicated space in PI Li’s lab and Co-investigator (CI) Lin’s lab, respectively, and kept in locked file cabinets with limited access. Other data including biomarker assessment are stored electronically with RedCap, a data management software, in the UMN Academic Health Center’s Information Systems (AHC-IS) servers. Both sites will have access to RedCap and use it for data storage and management. The AHC-IS servers are in a physically secure location on the UMN campus and are backed up nightly, with the backups stored in accordance with the AHC-IS retention schedule of daily, weekly, and monthly tapes retained for 1, 3, and 6 months, respectively. The AHC-IS servers provide a stable, secure, well-maintained, and high-capacity data storage environment that meets the requirements for storing even Health Insurance Portability and Accountability Act (HIPAA)-sensitive data. Access to the AHC-IS servers requires a username and password. PI Li works with the ACT Trial managers and lab technicians at both sites to audit the data quarterly to ensure accuracy and completeness of data collection and quality.

### Statistical analysis

The ACT Trial will share data on cognition, AD-signature cortical thickness, and other relevant information (e.g., demographics and medications) with this ancillary study for data analysis. We will perform appropriate transformation of cognition (episodic memory and global cognition) and AD-signature cortical thickness data. We will use Holm’s approach to adjust for the hypothesis testing of the multiple neuropathological, neurodegenerative, and neurotrophic biomarkers considered here, which will control the family-wise error rate [41]. Despite randomization, it is possible that the ACT Trial intervention groups could differ in important variables (e.g., age, education, medical comorbidities, medication use [collected by the ACT Trial] and *APOE* genotype). We will compare groups in terms of these variables and adjust for them if significant differences are found. Missing data due to missed collection visits, loss to follow-ups, and dropouts will be recorded and reported. Missing data will not be imputed. The inverse probability weighting method or likelihood-based method will be used for data analysis assuming the missing data are missing at random. Analyses with complete data or the use of other methods such as the mixture pattern model will be conducted to test the sensitivity of the results to the assumption of missing at random. Although our aims are not focused on age, sex, and *APOE* genotype, we will consider them as key covariates because they are established risk factors for AD [42]. Thus, we will adjust for these three variables as covariates in all analyses to test for significant associations using linear models.

#### Aim 1: determine the effects of interventions on blood biomarkers over 6 months in aMCI

To test *Hypotheses 1a* and *1b*, we will investigate the association between interventions and changes in blood neuropathological or neurotrophic biomarker levels over time. We will use separate linear regression models for testing each hypothesis with the 6-month changes of blood biomarker levels as the outcome variables. *Hypothesis 1b* is the primary hypothesis. The model for testing the primary hypothesis will include two binary indicators, for aerobic exercise and cognitive training, respectively, as the predictors to test the main effects of aerobic exercise and cognitive training. The model for testing *Hypothesis 1a* will include two binary indicators, for aerobic exercise and cognitive training, respectively, as well as their interaction term to test for the synergistic effect of these treatments on blood biomarker level changes, respectively. We will also include either age, sex, or a binary indicator for the presence of the *APOE* ε4 allele as covariates. The *p* values from these models will be adjusted across all biomarkers using Holm’s approach.

#### Aim 2: evaluate blood biomarkers as surrogate endpoints for predicting cognitive responses to interventions over 18 months in aMCI

To test the hypothesis in this aim, we will develop longitudinal linear regression models that investigate the association between changes in blood neuropathological or neurotrophic biomarker levels and cognitive changes over time in response to the interventions. These models will have the change from baseline for the cognitive responses as the response variable and will include ACT treatment arm, age, sex, presence of the *APOE* ε4 allele, baseline cognition, and a time-varying blood biomarker (e.g., change in a blood biomarker level for a participant between baseline and 3 or 6 months) as covariates. We will fit a model for each blood biomarker and use Holm’s method for multiple test adjustment. Any significant association between changes in cognition and in biomarker levels would be clinically useful because biochemical assays are much more sensitive to discern changes in blood biomarker levels than cognitive tests to discern changes in cognition for detecting cognitive treatment effects of interventions.

#### Aim 3 (exploratory): examine the correspondence between changes in blood and MRI biomarkers in response to interventions over 18 months in aMCI

The associations between blood and MRI biomarkers at each time point will be evaluated, and 95% confidence intervals (CIs) will be reported. In addition to this analysis, we will adjust for intervention arms as well as the key biological variables. We will also examine longitudinal linear regression models similar to those used for evaluation of the association between blood biomarkers and cognitive responses in Aim 2, which will test the associations after adjusting for covariates, including ACT treatment arm, age, sex, and presence of the *APOE* ε4 allele.

### Dissemination

The study findings will be disseminated through publications and presentations. The study will follow the established publication guidelines for authorship (e.g., the International Committee of Medical Journal Editors). The study does not intend to use professional writers. Access to the final trial data set will follow the guidelines of the NIA.

## Discussion

While we have carefully designed this study, we have anticipated potential problems and have identified alternative strategies. Our sample size estimation is based on both a 94% enrollment rate and the same attrition rates as the ACT Trial: 25% at 6 months, 30% at 12 months, and 35% at 18 months. Genetic testing and fear of needles are two general concerns that may deter study participants from enrollment in a blood biomarker study. We will train our staff with talking points so that they are prepared to alleviate these concerns should they come up in our study. We will closely monitor the enrollment rates at both study sites. We will review key benchmarks such as recruitment, enrollment, blood collection, and sample storage each year to identify challenges and solutions. In the event of lower enrollment rates than anticipated (i.e., 94%), we will learn critical information related to recruitment to guide future studies.

Lifestyle factors (e.g., diets, cognitive activity, and physical activity), medications, and vascular diseases may affect blood Aβ_42_, [7] as well as blood neurotrophic factors [28, 30]. For example, plasma Aβ_42_ is increased by hypertension, ischemic heart disease, diabetes, medications, and the *APOE* ε4 allele.

To control for lifestyle factors, we will conduct a blood-draw assessment before each draw to collect information on several factors, including, but not limited to, medications, infection, and vascular disease conditions, unsupervised physical activity, and strenuous cognitive activity outside the ACT Trial. We will investigate these factors in statistical models and control for them. Furthermore, because we will measure changes in biomarkers in comparison to baseline biomarker levels, each participant’s baseline values will serve as his/her own controls. This approach means that our data analyses will unlikely be affected by medications that are already used and lifestyle factors and conditions that are already manifested at baseline but remain unchanged across the 18-month study period. If medication surveys and the blood-draw assessments indicate that any medications, lifestyle factors, or conditions known to affect blood biomarkers have changed dramatically for a participant during the study, we will control for such changes in the data analyses.

Studies have shown that transient increases in the plasma neurotrophic biomarkers BDNF and IGF-1 observed following an exercise training session are more dramatic than exercise-induced increases in their resting levels [30]. However, studies have also shown that a program of exercise training also increased resting levels of these biomarkers, and that larger increases correlated with increases in hippocampal volume and memory [43]. It is challenging to incorporate collection of pre- and post-training blood samples into the ACT Trial’s current study design. In addition, because resting levels of neurotrophic factors reflect constant, not just transient, stimulation of neurogenesis, and we are interested in how blood neurotrophic factor levels would reflect constant stimulation of neurogenesis, we will evaluate these changes in resting levels of neurotrophic factors.

Unequal dropouts across the four arms (three interventions plus one attention control) may produce uneven distribution of sample sizes. The unequal dropouts would affect power calculation in Aim 1 but not in Aim 2, which is the main effect of biomarkers. Therefore, we took this into consideration when we calculated power for Aim 1 by assuming the difference in sample size between any two arms is no greater than 3. The uneven distribution of sample sizes across arms does not affect statistical analyses, as we include intervention as a covariate in the linear regression models.

### Trial status

The status of the cohort study at the time of manuscript submission is open for enrollment. We started enrollment in August 2018 and expect enrollment accrual to complete in May 2022. The manuscript is based on the study protocol version 4.01 (November 11, 2018).

## Data Availability

Not applicable*.*

## Abbreviations

ACT: Combined aerobic exercise and cognitive training
AD: Alzheimer’s disease
aMCI: Amnestic mild cognitive impairment
APOE: Apolipoprotein E
ARIC: Atherosclerosis Risk in Communities
Aβ_40_: Amyloid beta 40
Aβ_42_: Amyloid beta 42
BDNF: Brain-derived neurotrophic factor
C0: dl-carnitine
C5: Valeryl-l-carnitine
CI: Co-investigator
CSF: Cerebrospinal fluid
ELISA: Enzyme-linked immunosorbent assay
IGF-1: Insulin-like growth factor 1
IGFBP-3: IGF-binding protein 3
IRB: Institutional Review Board
MRI: Magnetic resonance imaging
NfL: Neurofilament light
NIH: National Institutes of Health
PI: Principal Investigator
p-tau: Phosphorylated tau
RCT: Randomized controlled trial
REDCap: Research Electronic Data Capture
SCAC: Short-chain acylcarnitine
t-tau: Total tau
UMN: University of Minnesota
UR: University of Rochester
